# Tuning the color of high-karat gold in Au-TiO_2_ nanoparticle composites all the way to black

**DOI:** 10.1016/j.isci.2024.109655

**Published:** 2024-04-01

**Authors:** Lidia Rossi, Endre Horváth, Tianyi Wang, Claudio Grimaldi, Andrzej Sienkiewicz, Bence Gábor Márkus, David Beke, László Forró

**Affiliations:** 1 Laboratory of Physics of Complex Matter, École Polytechnique Fédérale de Lausanne, Lausanne, Switzerland; 2 Stavropoulos Center for Complex Quantum Matter, Department of Physics and Astronomy, University of Notre Dame, Notre Dame, IN 46556, USA; 3 Laboratory for Quantum Magnetism, École Polytechnique Fédérale de Lausanne, Lausanne, Switzerland; 4 ADSresonances Sàrl, Martigny, Switzerland; 5 Institute for Solid State Physics and Optics, Wigner Research Centre for Physics, Budapest, Hungary

**Keywords:** Physics, Optics, Materials science

## Abstract

For centuries, artisans have harnessed gold nanoparticles to imbue their creations with the vibrant hues that captivate the eye through interactions with visible light. In modern times, these distinct optoelectronic characteristics have pivoted toward the forefront of innovative technologies, finding their niche in advanced applications from solar energy to medicine, overshadowing their artistic heritage. This investigation reimagines the utilitarian scope of gold by innovating the optical characteristics of gold-titania nanostructures. This allows for an expanded palette of colors that retain the value of the precious metal. We employ nanostructured TiO_2_ in a high-pressure-high-temperature sintering technique that stabilizes Au nanoparticles, thwarting coalescence, and Oswald ripening. Further refinement is possible by engineering TiO_2_ color centers through the introduction of oxygen vacancies and Ti^3+^ ions, which aid in creating an opulent high-karat black-gold, but preserve the mechanical attributes essential to the integrity and function of the final product.

## Highlights

• Fabrication of 18k gold composites exhibiting distinctive hues of black, red, and purple
• TiO_2_ nanowires gold nanoparticles–based composites for high-karat hard gold materials
• Two synthesis pathways for the precise property modulation of TiO_2_-Au composites

## Graphical abstract

**Figure:**
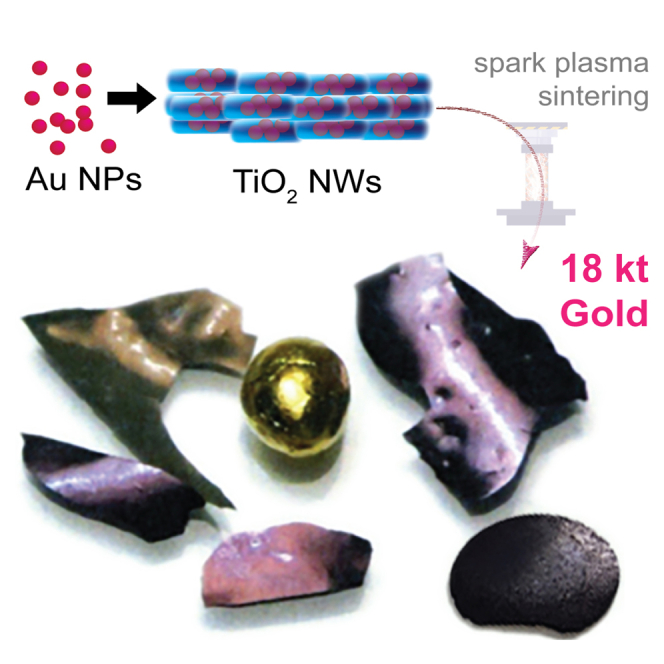

## Introduction

From the dawn of human civilization to the cutting-edge technologies of today, materials science has been an inevitable force in shaping society.^1^ It has empowered us to enhance living standards, simplify daily tasks, and even cultivate the beauty of art and jewelry. In contemporary times, one of the most exciting frontiers in this field is the development of nanostructured materials.^2^^,^^3^ These submicroscopic constructs are revolutionizing traditional material composites, offering unprecedented properties that find applications across a multitude of industries.

A common type of such composites is the hybrid nanocomposite, which involves dispersing nanoparticles into a macroscopic continuous matrix. Examples include carbon nanotubes or nanosized graphene flakes distributed in polymers, which significantly enhance the electrical and thermal conductivity of the matrix.^4^^,^^5^ Another interesting application involves gold nanoparticles (AuNPs) dispersed in a polydimethylsiloxane (PDMS) polymer, which allows to change the color of the material through the plasmon resonance effect modulated by variation in the distance between the particles stretching the polymer matrix.^6^ However, composites that consist only of truly nanosized materials are rather rare.

Gold is indeed a leading choice for jewelry^7^ and has diverse technological applications.^8^^,^^9^^,^^10^^,^^11^^,^^12^ Its iconic yellow hue can be altered by using various alloy combinations, such as mixing it with copper, silver, or platinum, resulting in the common gold shades called rose, white, and green.^13^^,^^14^ Special intermetallic phases like AuAl_2_ and AuIn_2_ can produce intense purple and blue hues, but these tend to be brittle and are more suitable for decorative purposes.^14^^,^^15^ Surface modification techniques, like oxidation, can also change the color of the surface depending on the alloy used.^13^^,^^14^

Unusual colors of karat gold, black in particular, have drawn plenteous interest in jewelry^14^^,^^16^^,^^17^^,^^18^ and technology.^11^^,^^12^^,^^17^^,^^19^^,^^20^^,^^21^ Surface coating^22^ and surface-nanotechnology solutions^16^^,^^17^ have shown promise in creating black gold; however, existing methods are expensive, limited to the surface, and yield products with limited durability.^16^^,^^17^^,^^22^^,^^23^ Despite the known distinct color of AuNPs, their use in decorations and jewelry, or in the production of materials having macroscopic extension in all dimensions is scarce. The two famous examples are the Alhambra palace’s purple color on the gold-gilded wall^24^ decoration resulted in corrosion-induced nanoparticle formation and the Lycurgus Cup.^25^

To overcome the current limitations in bulk-coloring of high karat gold, we have selected titanium dioxide (TiO_2_) as the nanostructured matrix for embedding AuNPs with a specific goal in mind: to develop bulk mixed gold-titania nanostructured composites that offer tunable karats (kt) up to 18 kt and color characteristics (red, violet, black, etc.). TiO_2_ has been the subject of extensive research due to its admirable thermal^25^ and chemical stability,^26^ minimal toxicity,^27^^,^^28^^,^^29^ and excessive natural abundance.^30^^,^^31^ Relative to other semiconductors, TiO_2_ holds a unique position for its adaptability across various applications,^32^ including but not limited to pollutant photodegradation,^33^ water-splitting for hydrogen production,^34^ and the formulation of self-cleaning coatings.^35^

Moreover, the color palette of TiO_2_ can be modulated from its innate white to a range of colors, including yellow, purple, blue, and black.^36^ This is achieved through the manipulation of color centers – specific point defects in its crystalline semiconductor structure.^37^ By skillfully merging AuNPs with titanium dioxide nanoparticles (TiO_2_NPs) and adjusting their intrinsic properties, we are enabled to fabricate gold composites exhibiting a diverse spectrum of colors, all while maintaining the requisite mechanical robustness and durability for daily use. The comprehensive understanding of the composite formation process allows us to engineer 18 karats gold materials with unconventional hues like black, red, and purple, which are incorporated *throughout the whole volume* of the material rather than being mere surface effects or coatings.

## Results and discussion

### What can we expect in tuning the color of gold? – Breakdown of the classical theory

**Figure 1:**
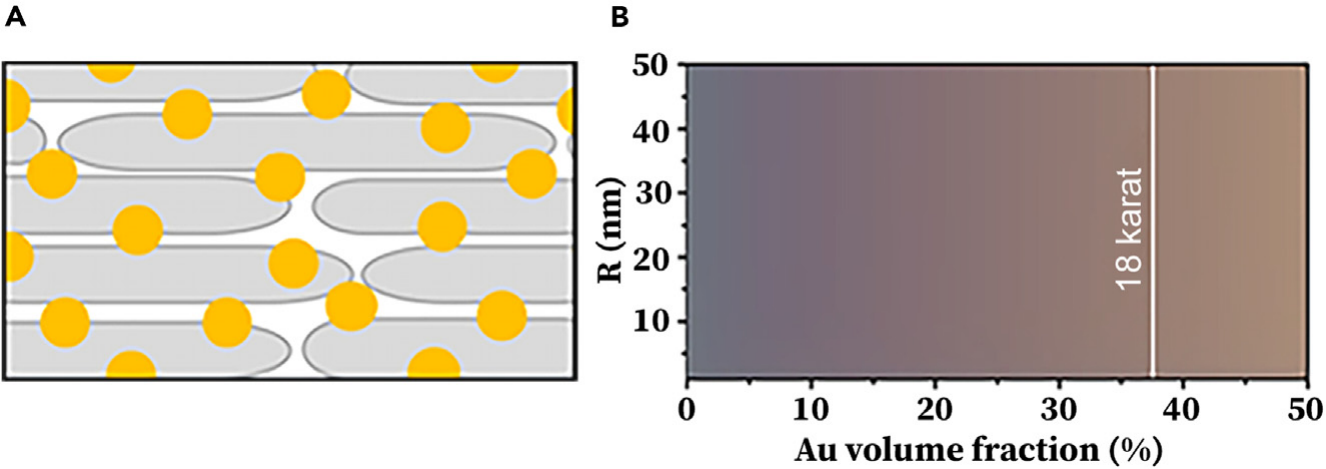
Theoretical model of TiO_2_/Au composites (A) Sketch of the TiO_2_NWs/AuNPs composite where TiO_2_NWs and AuNPs are marked by gray rods and yellow dots, respectively. By varying the concentration and size of AuNPs, one can tune the color of the composite by the plasmon resonance effect. (B) The Bruggeman effective medium approximation gives the corresponding range of colors (in reflection) as a function of volume fraction and radius of AuNPs. The blackish color corresponding to low f values indicates that virtually all incident light is absorbed with minimal reflection.

In the initial phase of our study, we focus on the theoretical possibilities of tuning the color of the TiO_2_/AuNPs composites. The tunability of color in TiO_2_ and the 'coloration' of Au NPs at the nanoscale hints a great promise. However, the optical properties of an upscaled nanosystem are difficult to predict from nanoscale results. The plasmonic effect gives AuNPs various colors ranging from rouge to purple depending on the particle-particle distance and particle size. Interestingly, a loss of this effect can occur when the particles are in very close proximity, causing the gold color to revert to its traditional hue. As the karat value increases, the particle-particle distance decreases. Therefore, we first performed theoretical calculations to determine if the manifestation of the plasmonic effect is possible at all at 18 kt. The schematic model is shown in Figure 1A. As depicted in Figure 1B, the size and volumetric concentration of AuNPs serve as the variable parameters. To model these aspects, we employ the Bruggeman effective medium approximation (EMA), which offers advantages over other mixing rules like the Maxwell-Garnett EMA, most notably its applicability beyond small filler loadings.^38^ Within the context of a two-phase composite, the Bruggeman EMA allows us to calculate the effective dielectric function of the composite, $\varepsilon_{\text{eff}}$. This is achieved by considering the complex dielectric function of the AuNPs (ε) embedded in a host medium. To get an insight we consider a host matrix of TiO_2_ Nanowires (TiO_2_NWs). However, the calculation itself is not restricted to this geometry. Other structures can be accounted by slightly varying the dielectric constant of the host, $\varepsilon_{m}$. In this case is a structure formed by an assembly of TiO_2_NWs with a specific dielectric function can be expressed as follows:

(Equation 1) $$f\frac{\varepsilon -\varepsilon_{\text{eff}}}{\varepsilon +2\varepsilon_{\text{eff}}}+\left(1-f\right)\frac{\varepsilon_{m}-\varepsilon_{\text{eff}}}{\varepsilon_{m}+2\varepsilon_{\text{eff}}}=0\text{,}$$

where f is the volume fraction of the *gold* phase in V/V% Au. In Equation 1, we assumed that the AuNP fillers are spherical in shape with a radius denoted by R. The dielectric function of these AuNPs is approximated using the Drude model,^39^ with corrections for finite-size effects. These corrections are essential for accounting for the shift in plasmon resonance attributable to the nanoscale dimensions of R.

Using the EMA dielectric function $\varepsilon_{\text{eff}}$ derived from Equation 1, we compute the reflectance of the bulk composite under incident sunlight. Subsequently, we determine the perceived colors based on both the AuNP radius R and the gold volume fraction f. As depicted in Figure 1B, the sizes of AuNPs, ranging from R=1 nm to R=50 nm, have minimal impact on the composite’s reflected color, irrespective of f. This phenomenon can be attributed to the fact that the wavelengths of visible light, which lie between 400 nm and 700 nm, are considerably larger than R, therefore, only Rayleigh scattering can occur, and the resonance frequency, or localized surface plasmon resonance, only changes by about 20 nm. This explains the size independence observed in the theoretical calculations. It is noteworthy that we considered a homogeneous TiO_2_ matrix in the calculation, whereas, in a real nanocomposite, the AuNPs are surrounded by TiO_2_ nanoparticles, leading to inhomogeneity and variations in the dielectric function of the medium. Nevertheless, our calculation reveals that we can maintain some of the colors of AuNPs even in the high-karat range.

### How can it be done? – Experimental approach to make gold colorful

**Figure 2:**
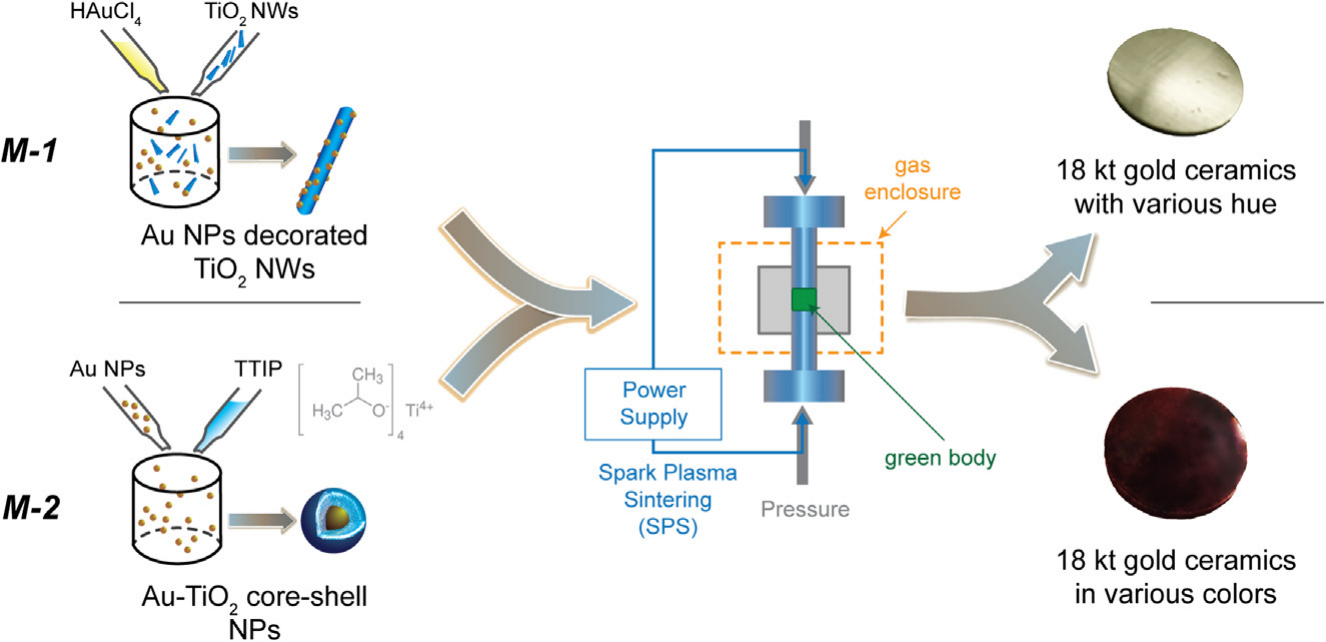
Schematic representation of the two technological routes employed for synthesizing TiO_2_/Au composites In the *M-1* approach, AuNPs are deposited onto pre-synthesized TiO_2_ nanowires (TiO_2_NWs). Conversely, in the *M-2* approach, AuNPs are encapsulated within a TiO_2_ shell. Following the drying of these respective powders, both undergo identical compacting and sintering processes.

We developed two unique types of composite ceramics: TiO_2_ nanowires (TiO_2_NW) encrusted with gold nanoparticles (TiO_2_NWs/AuNPs) and nold nanoparticles Coated with TiO_2_ (Au@TiO_2_ NPs). Each composite material showcases distinct optical properties and was crafted through a scalable technological route – referred to as Method-1 (*M-1*) for TiO_2_NWs/AuNPs and Method-2 (*M-2*) for Au@TiO_2_ NPs. Figure 2 provides a schematic overview of these synthesis pathways.

TiO_2_ Nanowires, crucial for the TiO_2_NWs/AuNPs composite via the *M-1* pathway, were fabricated in our laboratory using an advanced large-scale synthesis method as depicted in Figure 2. For an in-depth understanding of TiO_2_NWs synthesis, we refer readers to.^40^^,^^41^^,^^42^ Gold nanoparticles (AuNPs), integral to both Method-1 and Method-2, were synthesized through the reputable Turkevich method. This approach entails reducing chloroauric acid (HAuCl_4_) with citric acid (HOC(CH_2_CO_2_H)_2_) in a water-based medium, as delineated in. ^43^^,^^44^^,^^45^ The resultant AuNPs exhibit size dimensions ranging between 5 and 20 nm. Table 1 specifies the targeted concentration balance of TiO_2_NWs and AuNPs for various karats for TiO_2_NWs/AuNPs composite obtained in the *M-1* technological route.

**Table 1:** Relation between karats and composition

Karat	Au (wt%)	TiO_2_ (wt%)	***ρ*** (g/cm^3^)
18	75	25	9.7
16	66.7	32.3	8.4
14	58.3	41.7	7.2
12	50	50	6.5
1	4.2	95.8	4.0

**Figure 3:**
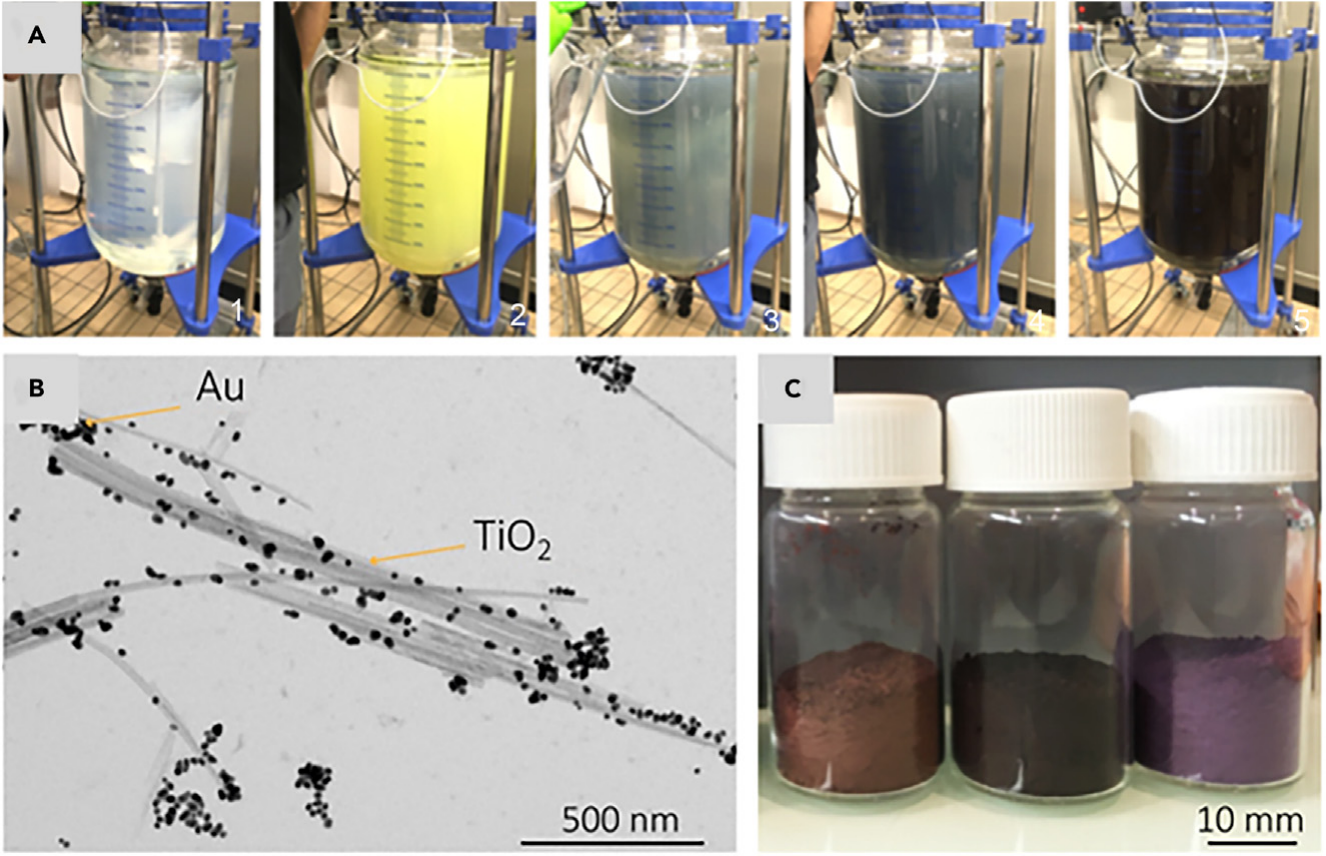
Fabrication of TiO_2_/Au composites nano-structural composites using the *M-1* technological route - from the building elements of gold/titania composite to their large-scale assemblies (A) Photographs of the chemical reactor for the synthesis of the TiO_2_NWs/AuNPs composite (1.: TiO_2_NWs suspension, 2.: after HAuCl_4_ was added, 3–5.: AuNPs growths). (B) TEM image of the 12 kt AuNPs and TiO_2_NWs after the liquid phase preparation. (C) Photograph of TiO_2_NWs/AuNPs composite powders prepared for the sintering process. From left to right: 18, 1, and 12 karats, respectively.

In particular, the fabrication of TiO_2_NWs/AuNPs nanocomposites via the Method-1 route was executed in a 100 L reactor containing distilled water maintained at 85°C, as illustrated in Figure 3A. Initially, TiO_2_NWs were dispersed in a minimal volume of water using an ultrasonic homogenizer/mixer. This suspension was then added to the reactor’s distilled water. Subsequently, the pH was adjusted, followed by the introduction of HAuCl_4_·3H_2_O and citric acid. After a reaction time of about 6 h, the material settled, allowing for sedimentation. The collected material then underwent a series of filtration, washing, and grinding processes, ultimately yielding powders composed of micron-sized grains. The transmission electron microscopy (TEM) image demonstrates the successful decoration of TiO_2_NWs by AuNPs (Figures 3B and S1). In addition, Figure 3C displays the representative TiO_2_NWs/AuNPs powders of different karats obtained through this method.

**Figure 4:**
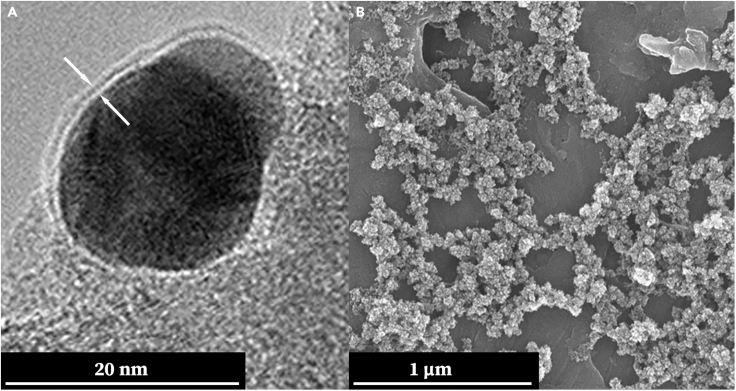
Presenting the key imaging data to elucidate the morphology of Au@TiO_2_ NPs synthesized via the (*M-2*) approach (A) Featuring a High-Resolution Transmission Electron Microscopy (HR-TEM) image of a 20 nm AuNP following the TiO_2_ shell deposition. The image clearly illustrates the thin TiO_2_ layer enveloping the AuNP core, captured at the edge of a holey carbon grid, as indicated by the arrows. (B) Displaying a scanning electron microscopy (SEM) image of the Au@TiO_2_ particles prior to the sintering process. This offers a comprehensive view of the particle ensemble, serving as a baseline for evaluating subsequent changes post-sintering.

For the synthesis of Au@TiO_2_ NPs composites using the Method-2 (*M-2*) approach, AuNPs of either 5 nm or 20 nm in size were initially dispersed in isopropanol. The pH of the mixture was then adjusted to a mildly basic level (pH = 9) using aqueous ammonia. Subsequently, Titanium (IV) isopropoxide (Ti[OCH(CH_3_)_2_]_4_) was added incrementally, triggering the hydrolysis reaction. This resulted in the formation of a TiO_2_ shell directly on the surface of the AuNPs. Upon completion of the TiO_2_ formation, the Au@TiO_2_ NPs were isolated by evaporating the solvent. These particles were then re-dispersed in aqueous ammonia, followed by stirring at 80°C for 24 h, using procedures consistent with the TiO_2_NWs synthesis as in.^40^^,^^41^^,^^42^ High-Resolution Transmission Electron Microscopy (HR-TEM) imagery capturing the structure of a single core-shell Au@TiO_2_ NP is presented in Figure 4A. A Scanning Electron Microscopy (SEM) image displaying a broad collection of these core-shell Au@TiO_2_ NPs is provided in Figure 4B.

To create materials amenable to further processing, the powders resulting from both methods were subjected to compaction using a closed-die press. This step promotes direct inter-particle contacts, reduces voids, and enhances the density and the hardness of the material. In metallurgical jargon, this initial cold-forming stage results in what is termed a 'green body'. The dimensions of these green bodies varied, ranging in diameter from 12 to 60 mm and in thickness from 0.7 to 2 mm.

Following powder compaction, the subsequent stage entailed high-temperature sintering using the spark plasma sintering (SPS) technique. The green body was positioned between electrically conductive metallic pistons, separated by graphite foils, to ensure optimal electrical and thermal contact. A current of 600 A at a voltage of 5 V was applied for the sintering process. Joule heating elevates the temperature of the sample during this stage, while solid-state diffusion facilitates inter-particle bonding. The sintering duration was intentionally capped at 20 min to mitigate the risk of excessive AuNP migration and coalescence.

**Figure 5:**
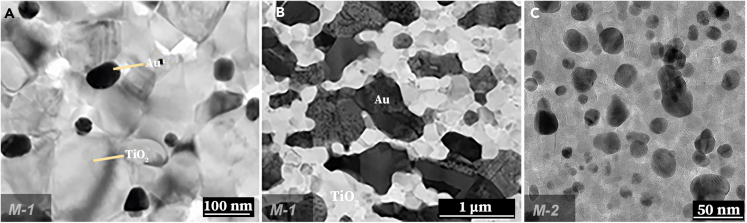
Transmission electron microscopy (TEM) images of the composites synthesized via the *M-1* or *M-2* technological routes after sintering (A) a 1 karat sample containing 4.2 wt% Au, (B) an 18 karats sample containing 75 wt% Au. Notably, in the 18 karats sample, there is visible coalescence of the AuNPs. In contrast, coalescence is not observed in the TEM images of the Au@TiO_2_ composites prepared via the *M-2* technological route after sintering, as shown in part (C) for the 18-karat ceramics. Although the particle sizes appear slightly larger than those seen in the high-resolution TEM (HR-TEM) images of Au@TiO_2_ samples prior to sintering and annealing, the crystallite dimensions remain unchanged. This stability is attributed to the protective TiO_2_ shell enveloping the surface of the AuNPs.

Throughout this high-temperature treatment, the original TiO_2_NWs undergo a transformation, collapsing into nanoparticles primarily in the rutile phase. TEM images showcasing the post-sintering morphology of samples from Method-1 are provided in Figures 5A and 5B. These images feature both 1 karat and 18 karats samples (see Figure S1 for more details).

The TEM images offer a clear distinction between the outcomes of the two synthesis methods, especially regarding nanoparticle coalescence. In the low-karat samples synthesized by *M-1*, the AuNPs are well-separated and maintain regular shapes, as shown in Figure 5A. However, coalescence becomes a pronounced issue in the high-karat, or 18 karats, samples, making the *M-1* route less suitable for maintaining the original sizes of AuNPs after sintering, as illustrated in Figure 5B.

This led us to develop an alternative approach, Method-2 (*M-2*), designed specifically to preserve the size and shape of AuNPs during sintering, especially in high-karat, jewelry-grade samples. Unlike *M-1*, which involves synthesizing AuNPs in the presence of TiO_2_ nanowires, *M-2* features a reverse process: TiO_2_ is synthesized to form a shell around pre-existing AuNPs. This protective TiO_2_ shell effectively mitigates the coalescence issue. Figure 5C confirms that *M-2* successfully retains the original sizes of the AuNPs after both sintering and annealing, alongside the presence of TiO_2_ nanoparticles. Size distribution results can be found in Table S1 and Figure S2.

### Gilded elegance: Unveiling the stunning aesthetics and comfort of synthesized gold samples

**Figure 6:**
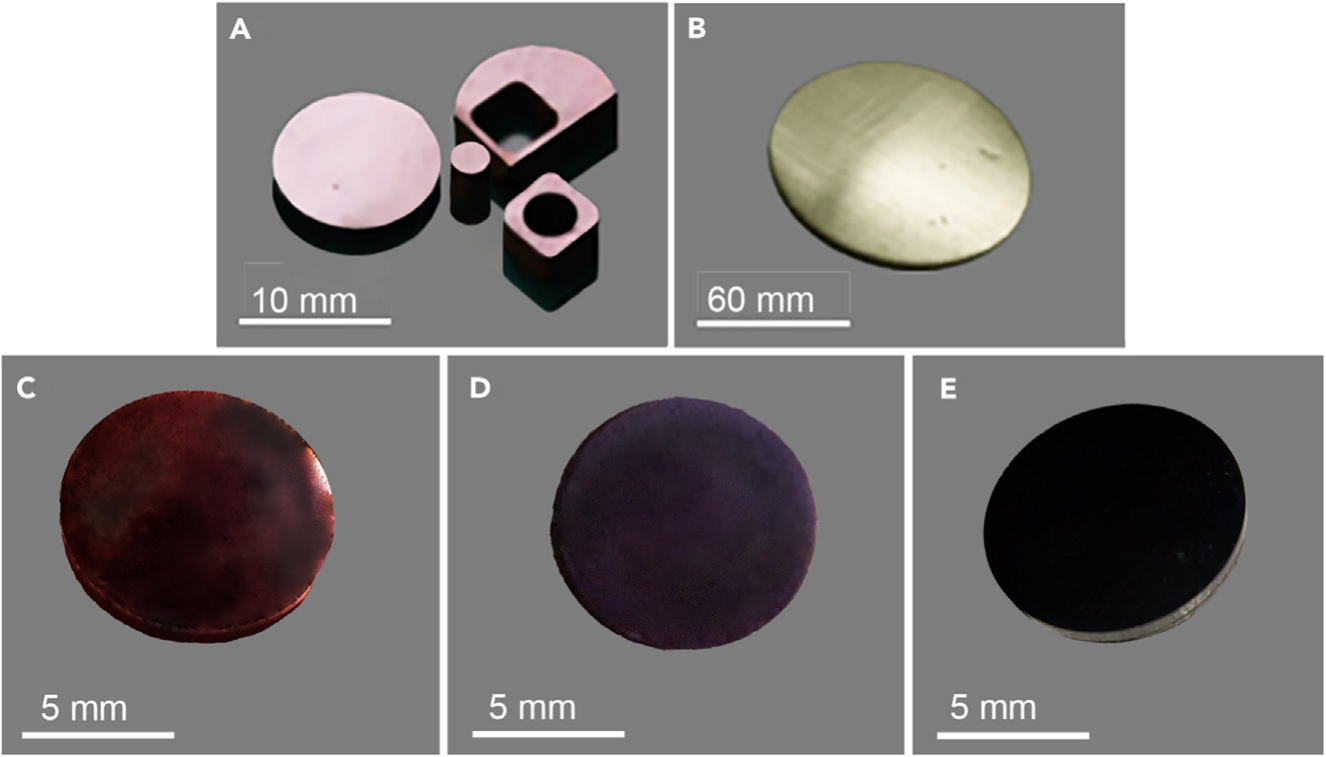
Photographs of the prepared Au-TiO_2_ composites Five representative ceramic discs of 12-karat (A), and 18-karat (B), samples produced using the *M-1* technological route, 18-karat samples made by *M-2* from 5 nm Au NPs (C) and 20 nm AuNPs sintered in air (D) and in 5% H_2_/Ar atmosphere (E) showing, brownish pink, khaki greenish, reddish, purple, and black colors, respectively. The image of the 12-karat sample (A) also exhibits the possibility of shaping this material into various forms by laser-assisted cutting.

Figure 6 displays photographs of the sintered ceramic discs, representing various karat weights such as 18-karat and 12-karat samples. These samples were prepared using two different synthesis methods, designated *M-1*, and *M-2*. To ensure a high-quality surface finish, all samples underwent a thorough dry polishing process employing a range of silicon carbide (SiC) abrasive papers with differing grit sizes.

Apparently, some of the produced TiO_2_NWs/AuNPs composites exhibit a broader color palette than expected by classical theory. The 12-karat sample from *M-1* has an intense pink color, while the 18-karat sample is golden with a khaki-green shade after atmospheric sintering; the colors can be darkened by sintering the samples under a reducing atmosphere. In contrast, samples from *M-2* exhibit a dark color corresponding to the AuNP size when sintered under air and turn utterly black when a reducing atmosphere is applied.

We hypothesized that this variation arises from two factors not accounted for in Equation 1. First, the classical theory may break down due to imprecisely treated plasmonic excitations. Second, color centers in the TiO_2_ matrix, which are not incorporated into Equation 1, could also contribute to the observed variations in color. Indeed, oxygen off-stoichiometry in TiO_2_ resulting from high-temperature treatment,^46^ and oxygen vacancies in TiO_2-*x*_ give rise to distorted octahedral sites of Ti^3+^ with an electron spin magnetic moment^47^ (S = ½). Even in single crystals, these defects create shallow donor states, which can render the material conducting over a broad temperature range and, consequently, completely black. This is a significant advantage of the TiO_2_-based matrix, which can tune the color of the composite material beyond the plasmonic effect. These color centers alter the appearance of TiO_2_ and, consequently, of the entire ceramic.

The influence of the sintering atmosphere on defect concentration and, consequently, on the color of the composite material, is fascinating. In materials science, the sintering atmosphere is known to affect phase transformations, defect concentrations, and mechanical properties. Here, its role extends to adjusting the optical characteristics of the nanocomposites, which could be of tremendous significance for applications where coloration and appearance matter, such as in jewelry or decorative coatings. The parameters that influence the color of the composites are summarized below.

(1) Reducing Atmosphere: When the green body is sintered in a reducing atmosphere like H_2_/Ar (5% H_2_), black TiO_2_ forms. This change dramatically alters the optical characteristics of the composite, enabling darker colors and even a black appearance, as demonstrated in the 18 karats TiO_2_NWs/AuNPs composites made through *M-2.*
(2) Oxidizing Atmosphere: On the other hand, sintering in an air (oxygen-rich) atmosphere has the opposite effect, lightening the color of the composites. In 18 karats samples obtained through *M-1*, the color can even turn to white-gold.
(3) Particle Size and Core-Shell Interactions: The color also seems to be influenced by the size of the gold core-shell nanoparticles, as shown by the 5 nm particles displaying a dark red color and the 20 nm particles showing a purple color when air-sintered.
(4) Complex Interplay: The final coloration appears to be a complex function of both the sintering atmosphere and the initial material properties, including gold concentration and particle size.

This ability to finely tune the composite coloration through the choice of sintering atmosphere, coupled with the choice of synthesis method (*M-1* or *M-2*), adds another layer of controllability to these nanocomposites. It offers intriguing possibilities for customized materials with specific optical or even electronic properties.

**Figure 7:**
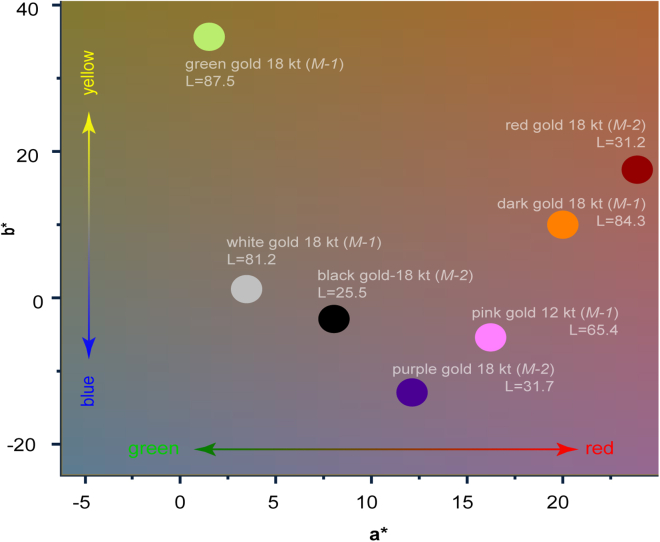
2D CIELab representation of the color of various karat gold samples The legend marks the karat, the production route, and the perceptual lightness (L) value.

The use of the industry-standard CIELab color system in our study adds a quantitative and standardized measure^48^^,^^49^ to the evaluation of the AuNPs composites. This is excellent for making cross-comparisons and is particularly useful for industrial applications where precise color matching is essential. This representation of color is quite similar to the Y’UV color space widely used in the motion picture industry.^50^^,^^51^ In the CIELab system, the three parameters (*L* for lightness, and *a*^∗^ and *b*^∗^ for the color opponents red-green and yellow-blue, respectively) create a multidimensional space and can objectively compare how different processing conditions, such as the *M-1* or *M-2* technological routes and the sintering atmosphere, affect the final color of the composites (Figure 7). It will be particularly insightful to see how these objective measures of color relate to changes in other properties like defect concentrations or crystallite sizes.

In the context of the jewelry industry, not only is the aesthetic appeal of color vital, but porosity and Vickers hardness also play crucial roles in determining the quality of sintered ceramics.^52^ The measurement of porosity in these ceramics can be accurately gauged through the application of the Archimedean principle. An array of tests conducted on a variety of samples has revealed that porosity is closely linked to the specific conditions under which sintering occurs, as well as to the gold content within the ceramic. Notably, the data indicate that at any specified gold concentration, there is a trend of diminishing porosity with the increase of sintering temperatures. The ceramics subjected to the most intense sintering temperatures exhibit porosities that are less than 2%, which underscores the effectiveness of high-temperature treatment in producing denser materials. On the other hand, ceramics with the highest gold content, particularly those with 18 karats gold, display greater porosity relative to their lower-karat counterparts. This variation is a pivotal consideration for the jewelry sector.

Turning our attention to hardness, this attribute was meticulously assessed using a state-of-the-art digital microhardness tester. The obtained Vickers hardness values are systematically collected in Table 2. There emerges a clear pattern where an increase in gold content correlates with a reduction in hardness. This softening effect is attributed to the corresponding decrease in the volume of TiO_2_ nanowires.

**Table 2:** Porosity and hardness of six samples sintered via SPS

Sample (Karat)	Synthesis method (*M-*)	Porosity (%)	Micro hardness (HV)
18	*1*	0.10±0.05	320±8
18	*2*	0.80±0.10	190±20
16	*1*	0.10±0.05	330±10
14	*1*	0.10±0.05	350±10
12	*1*	0.10±0.05	350±10
1	*1*	0.14±0.05	820±5

Recognizing the requirements of the jewelry industry for materials with reduced porosity and enhanced hardness, we are optimistic that through dedicated optimization of the sintering processes, such improvements are attainable. It is within our technological grasp to fine-tune these parameters to suit the existing standards demanded by jewelry connoisseurs and manufacturers alike.

### What is the physics behind the obtained colors?

In order to comprehend the extensive color tunability of the synthesized composites and to test our hypothesis, a thorough analysis was conducted utilizing High-Resolution Transmission Electron Microscopy (HR-TEM), Scanning Electron Microscopy (SEM), X-ray Diffraction (XRD), Electron Spin Resonance (ESR), and Raman spectroscopy. This analysis was primarily focused on discerning the impact of synthesis methods on the size, morphology, and defect composition of AuNPs and the TiO_2_ matrix, and their subsequent influence on the optical properties of the gold samples prepared. XRD, SEM and TEM provide information of the particle, or crystallite size and size distribution with the addition particle shapes from TEM and SEM images (see Figures S1 and S2; Table S1). XRD and Raman data unhide the crystalline phases together with the various defects in such phases, while ESR and Raman spectra can be used to study paramagnetic defects.

**Figure 8:**
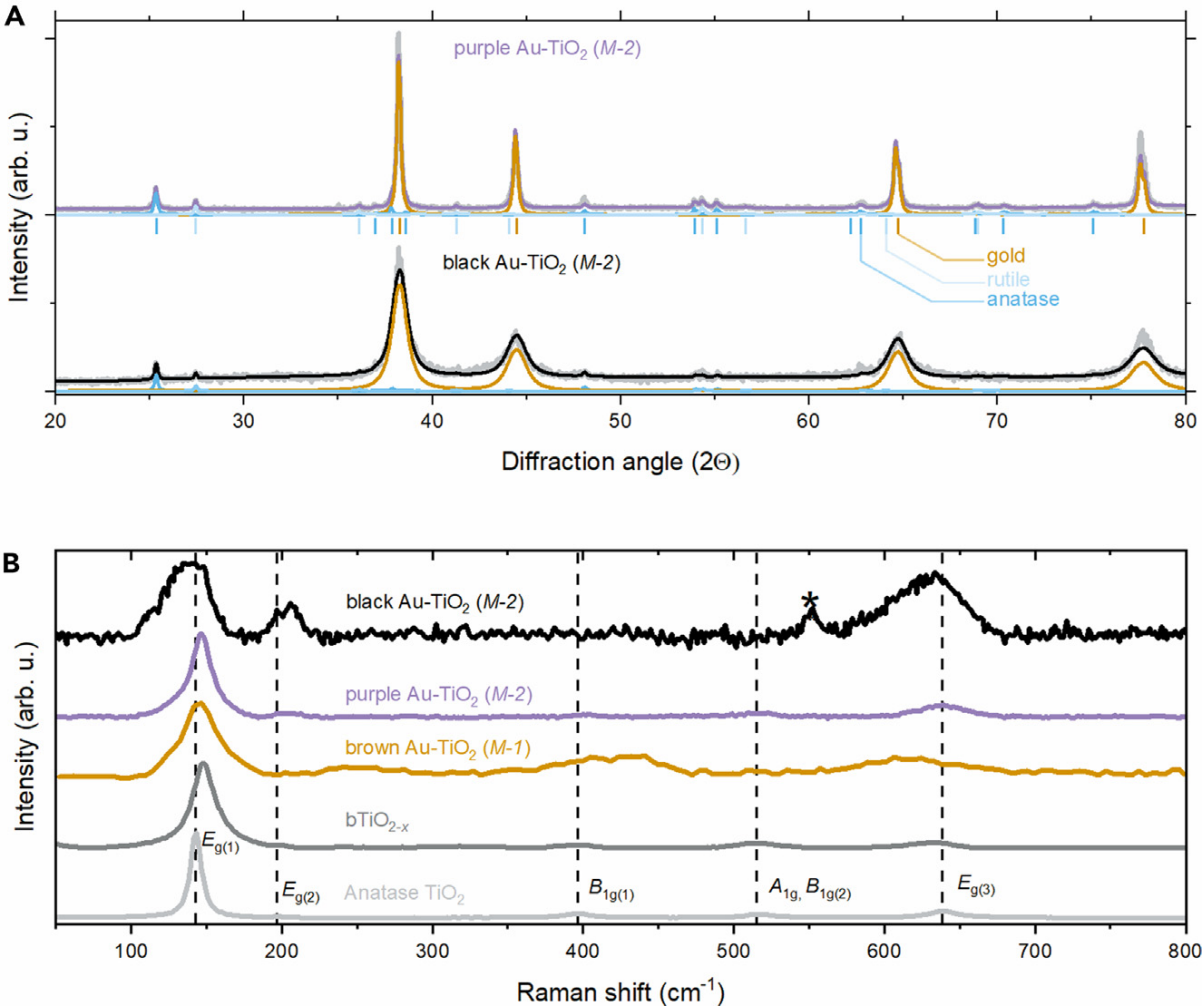
Materials characterization (A) XRD and (B) Raman spectra of various 18-karat composite ceramics obtained by the *M-1* and *M-2* technological routes. The powder X-ray diffractograms show the peak positions and resulting curves from Rietveld full profile analysis, which gives the crystallite size and concentrations of the components. The Raman-active irreducible representations are labeled and marked with vertical dashed lines. The main peak corresponding to the brookite phase in black gold sample is labeled with an asterisk. The other peaks are close to that of the rutile and anatase phases. A detailed description of the observed features can be found in the Supporting Information.

The structural investigations demonstrate substantial growth of AuNPs in the *M-1* synthesized samples at higher karat levels (see Figures 5 and S1), elucidating the origin of the gold color’s various tints in the 18-karat samples. The combined insights from XRD, Raman (Figures 8 and S3), and ESR data (Figure S4; Table S2) confirm the presence of the postulated point defects within the TiO_2_ matrix, accounting for the observed color variations. In particular, strain along with point defects in the brookite phase, were identified. The interface of Au and TiO_2_, along with the thermal treatment during synthesis, fosters these structural changes,^53^ which are contingent on the dimensions of the gold particles. Such interplays may also be leveraged to further refine the properties of the composite, potentially enhancing self-cleaning capabilities through the strain and point defects of the brookite phase of TiO_2_.^35^^,^^54^ Comprehensive results from the XRD, ESR, and Raman analyses are documented for further review in the supplemental information.

### Conclusions and outlook

The successful development of an innovative composite material, incorporating nanoscopic gold (Au) and titanium dioxide (TiO_2_) particles, marks a notable advance, paving the way for mass production scalability. By harnessing the intricate interplay of plasmonic phenomena, quantum effects, and color centers within TiO_2_, the color spectrum of 18 karats gold has been remarkably broadened beyond its iconic yellow to include a sophisticated black. This avant-garde bulk ceramic is poised to capture the interest of various sectors, notably watchmaking and jewelry, which are perennially on the quest for distinct and premium alternatives to traditional materials. Furthermore, the adaptability of these composites to be seamlessly shaped into intricate jewelry designs using conventional techniques — ranging from mold-sintering and forging to machining — augments their attractiveness. Coupled with self-cleaning capabilities and robust color retention, these novel materials stand out as particularly suited for high-end jewelry and luxury goods.

The potential of these materials transcends mere aesthetic value. The unique black surface, introduced by this composite, manifests significant utility across an array of technological domains. Such metallic black surfaces are instrumental for diverse applications: they enhance broadband absorption in photodetectors and thermal sensors, contribute to the precise monitoring of Earth’s radiation balance, improve the efficacy of solar energy collection, bolster catalytic processes, and facilitate efficient energy transfer in bio-electrochemical systems, among others. This expansive scope of applications underlines the transformative potential of this material innovation.

### Methods

**Scanning electron microscopy (SEM)** images were captured in vacuum using a FEI Magellan 400 microscope operated at 10 kV electrons at the NDIIF. Images were analyzed using ImageJ software.

**Transmission electron microscopy (TEM)** and **high-resolution transmission electron Microscopy (HR-TEM)** images of *M-1* samples were imaged using a FEI Talos microscope operated at 200 kV and a JEOL TEM 2200FS having a spatial resolution of 5 nm and a probe diameter of 2 nm. Images of *M-2* samples were captured on a Thermo Fisher Scientific Spectra 300 using 300 keV electrons at the NDIIF.

**Nanoindentation** measurements were performed with a Bruker Hysitron Nanoindenter 950 TI at the ASEND Core Facility of Notre Dame. Hardness values obtained through fitting the Force-Displacement curves using the TriboScan software.

**Raman** measurements were performed using a WITec alpha300R confocal Raman microscope using a Zeiss LD EC Epiplan-Neofluar 50×/NA = 0.55 objective and 1800 lines/mm grating. We used a 532 nm green laser at a fixed power of 0.5 mW as excitation. No physical damage was observed on the samples after the measurements. The obtained spectra were analyzed through Lorentzian fitting using the Levenberg-Marquardt least squares algorithm.

**XRD** spectra were obtained using a Bruker D8 Discover high-resolution spectrometer using the Cu K*α* line in the *θ*−2*θ* gometry. Rietveld refinement was carried out using the Profex software.

**ESR.** Low-temperature (5 K) continuous-wave (CW) ESR measurements were carried out with a Bruker ESR spectrometer E500 Elexsys Series (Bruker Biospin GmbH) equipped with a Gunn diode-based CW microwave bridge (model SuperX), a Bruker ER 4122 SHQE cavity and an Oxford Instruments Helium-gas continuous flow cryostat (model ESR900). For all measurements a conventional field-modulation technique combined with lock-in detection was used, which provided the first derivative of the ESR absorption spectra.^55^

The typical instrumental settings were the following: microwave frequency ∼9.42 GHz; microwave power 200 μW; magnetic field modulation frequency and amplitude of 100 kHz and 3.0 G, respectively.

### Limitations of the study

Due to the small quantity and fast hydrolyzation of Titanium (IV) isopropoxide, getting the exact TiO2-gold ratio in high-carat samples for small batches is difficult. We found a variation in the karat value of +/1 1 when 20 mg Au NPs were used.

## STAR★Methods

### Key resources table

**Table:** 

REAGENT or RESOURCE	SOURCE	IDENTIFIER
**Chemicals, peptides, and recombinant proteins**		

Tetrachloroauric(III) acid trihydrate 99%	Sigma Aldrich	G4022
Citric acid	Sigma Aldrich	251275
Titanium(IV) oxide, anatase	Sigma Aldrich	248576
Titanium(IV) isopropoxide	Sigma Aldrich	8.21895

**Software and algorithms**		

OriginLab OriginPro 2023b	OriginLab Co.	https://www.originlab.com/
Profex 5.1.1		https://www.profex-xrd.org/

### Resource availability

#### Lead contact

Further information and requests for resources and reagents should be directed to and will be fulfilled by the lead contact, David Beke dbeke@nd.com.

#### Materials availability

This study did not generate new unique reagents.

#### Data and code availability

Any additional information required to reanalyze the data reported in this paper is available from the lead contact upon request.

Data

• All data reported in this paper will be shared by the lead contact upon request.

Code

• This paper does not report original code.

### Method details

#### Synthesis of TiO_2_ NWs

Either potassium hydroxide (KOH) or sodium hydroxide (NaOH) is dissolved in 100 L of water to achieve a solution with a pH of 12. Into this alkaline solution, a titanium-containing precursor, specifically TiO₂ anatase, is introduced. The mixture is then heated to 70°C in a base-resistant vessel. To facilitate the formation of a high fraction of mesoporous titanates, shear mixing is applied for 24 hours using a brush-like stirrer at a speed of 1000 rpm. Following the synthesis, the resultant titanium oxide material, exhibiting a jelly-like consistency, is transferred to a centrifuge to separate the synthesized product from the residual solvent. Subsequently, the pH of the sample is adjusted to neutral (pH = 7) through repeated washing with water. This pH adjustment is instrumental in facilitating the formation of TiO₂ nanowires.

All the other preparations can be found in the main text in details.

#### Raman spectra evaluation

The three TiO_2_ phases (anatase, rutile, and brookite) have similar irreducible representations. Anatase has five Raman active modes *E*_g(1)_ at 143 cm^-1^, *E*_g(2)_ at 197 cm^-1^, *B*_1g(1)_ at 396 cm^-1^, *A*_1g_,*B*_1g(2)_ at 515 cm^-1^, and *E*_g(3)_ at 638 cm^-1^. Similar values can be found for the rutile^56^ and the brookite^57^^,^^58^ phases as well. The most intense peak is the *E*_g(1)_ in the anatase phase,^56^^,^^59^ similarly to the studied samples. The Raman spectra, however, can indicate the presence of vacancies and other dislocations,^37^^,^^60^ size^54^ strain,^53^ and other disturbances in the crystal structure. All spectra were fitted with Lorentzian curves to determine the peak positions and FWHM values. The difference between the anatase and the bTiO_2-*x*_ peak is that the peak position in bTiO_2-*x*_ is shifted from 144 cm^-1^ to 147 cm^-1^ and the FWHM is gradually increased from 9 cm^-1^ to 17 cm^-1^. The result of the evaluation shows tensile strain within the materials,^53^ most pronounced in the black gold variant characterized by the smallest Au particles and a core-shell configuration. The strain, along with point defects in brookite TiO_2_, is implicated in inducing phase shifts and peak broadening within the 200-800 cm^-1^ range, particularly noticeable in the 18-karat *M-1* samples. The interface of Au and TiO_2_, along with the thermal treatment during synthesis, fosters these structural changes,^53^ which are contingent on the dimensions of the gold particles.

#### Electron Spin Resonance spectroscopy

Electron spin resonance (ESR) was employed to precisely determine the concentration of paramagnetic centers in parts per million (ppm) in the samples sintered at high temperature. With the growth of the sintering temperature,the 1-karat sample demonstrates a significant increase in the signal of paramagnetic Ti^3+^ centers as compared to the material not subjected to heat treatment (Table S2), leading to higher electrical conductivity. In fact, the sample exhibits a resistivity as low as 3.2×10^−2^ Ω·cm at room temperature, displaying metallic temperature dependence down to 100 K. This metallic behavior enables broad photon absorption in the sample. In contrast, the 18-karat material exhibits metallic behavior down to 10 K with a resistivity of 1.8×10^−4^ Ω·cm at room temperature. A similar effect likely influences the color of higher karat samples.

A representative ESR spectrum obtained at 5 K, together with its fit are shown in Figure S4 for a 1-kt sample sintered at 900°C.
