# Supplementary material for: Tuning the color of high-karat gold in Au-TiO2 nanoparticle composites all the way to black
Source: iScience. 2024 Apr 1;27(5):109655. doi: 10.1016/j.isci.2024.109655 (PMC11068548; doi:10.1016/j.isci.2024.109655)
Supplement: Document S1. Figures S1–S4 and Tables S1 and S2 [file mmc1.pdf]

iScience, Volume *27*

Supplemental information

Tuning the color of high-karat gold in Au-TiO_2 nanoparticle composites all the way to black

Lidia Rossi, Endre Horváth, Tianyi Wang, Claudio Grimaldi, Andrzej Sienkiewicz, Bence Gábor Márkus, David Beke, and László Forró

Supplementary Materials

S1. Particle size characteristics

Figure S1 and **Table S1** provide a comparative summary of crystallite sizes before and after sintering for both synthesis methods.

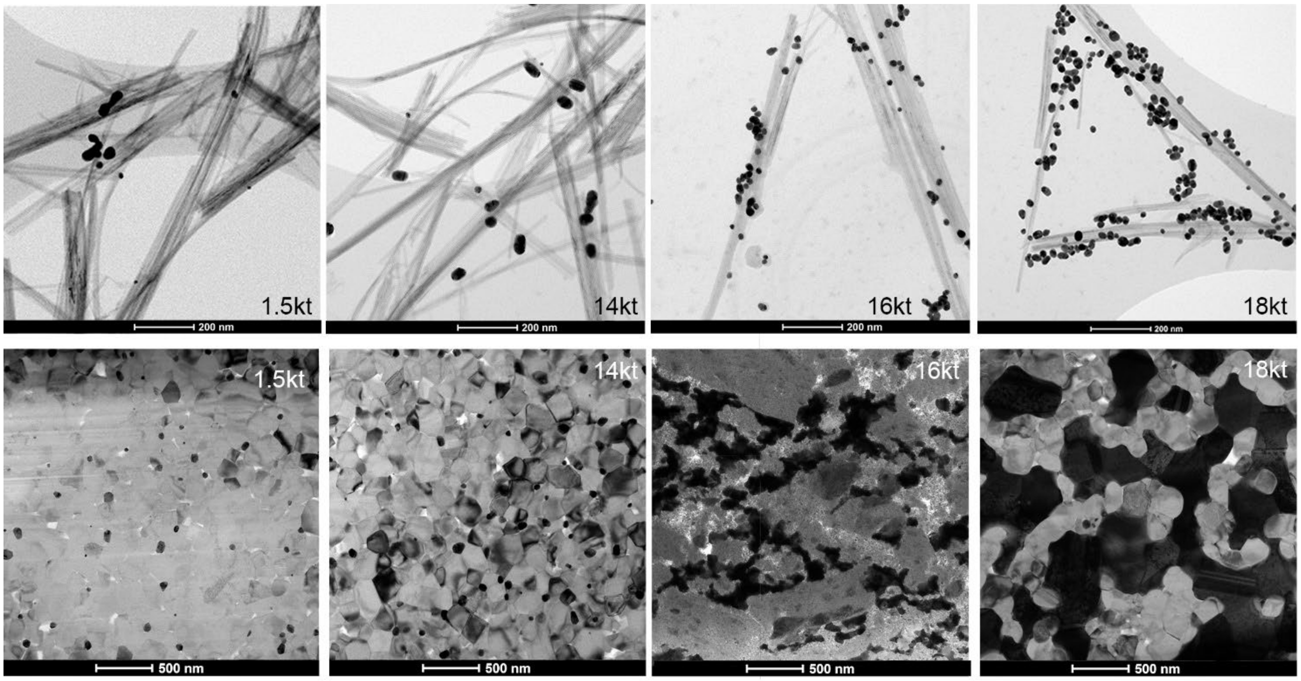

Figure S1: TEM images of various karat gold composites at different stages of the synthesis process, Related to Figure 3 and Figure 5. TEM images were taken after reaction (Method 1, top) and after sintering (Method 1, bottom). The sintered composites were cut and thinned with FIB for TEM analysis.

Table S1. Crystallite sizes of TiO_2NWs and AuNPs in select composite ceramics after the sintering process from XRD Rietveld analysis related to Figure 8 and Figure S3. The initial size of the AuNPs is indicated in square brackets, followed by the post-sintering size with the associated standard deviation. data.

Sample (Karat)	*AuNP* [original size] nm	*TiO_2NPs* nm	*Method*
18	[20] 44.5 $\pm$ 0.6	24 $\pm$ 1.7	*M-2*
18	[5] 9.4 $\pm$ 0.3	24 $\pm$ 1.7	*M-2*
18	[20] 100-600 $\pm$ 30	200 $\pm$ 32	*M-1*
16	[20] 52.8 $\pm$ 0.8	42.4 $\pm$ 1.3	*M-1*
14	[20] 32.5 $\pm$ 0.9	42.4 $\pm$ 1.6	*M-1*
12	[20] 31.8 $\pm$ 0.8	24.5 $\pm$ 1.2	*M-1*
1	[20] 30 $\pm$ 0.8	60 $\pm$ 2.1	*M-1*

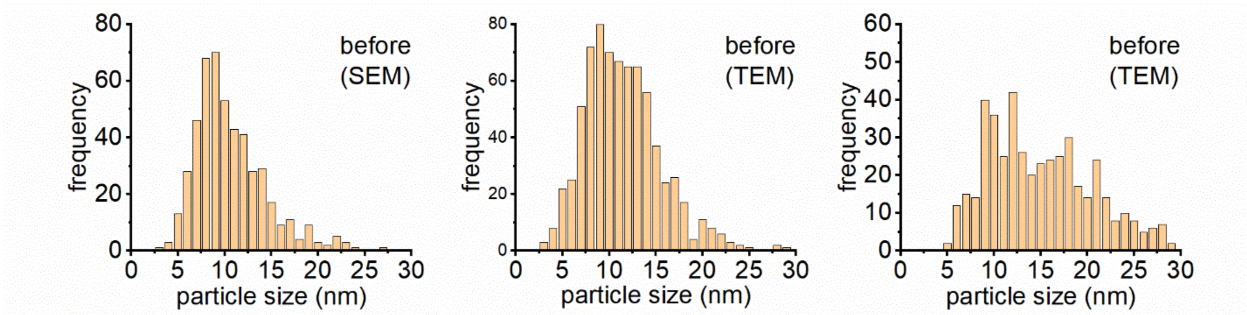

Figure S2: Au particle size distributions of 18 kt gold composite related to Figure 3 and Figure 5. The frequency spectra calculated from measurements of particle sizes before and after sintering the 18 kt samples made by the method *M-2.*

S2. Structural characteristics

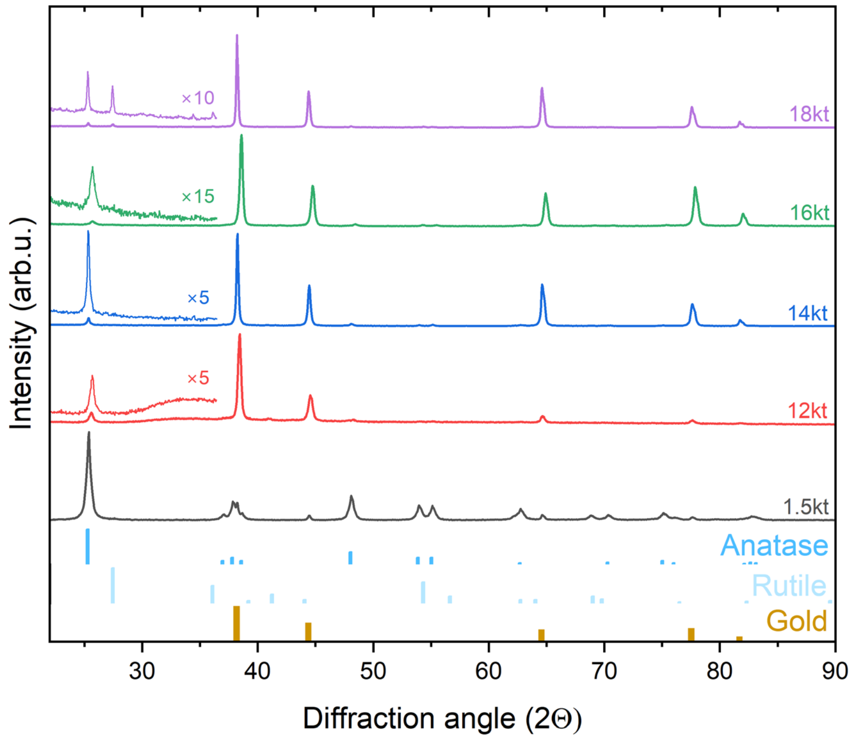

Figure S3: Powder X-ray diffractogram of the composite materials made via Method-1 Related to Figure 8. Labels indicate the karat value. Upper lines are the low-angle diffractogram magnified by a factor noted on the plot.

S3. Electron Spin Resonance (ESR) and resistivity measurements

Table S2: Spin concentrations in 1 kt samples related to STAR Method.

Sintering temperature (°C)	Spin concentration (ppm)
900	421
1100	485
1300	865

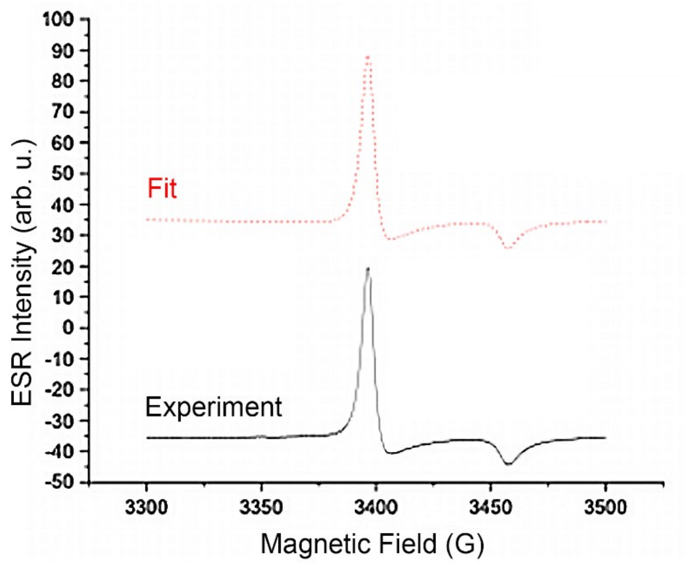

Figure S4: ESR spectrum of a 1-kt sample related to STAR Method. The measurement temperature was 5 K. The sample was prepared with the M-1 route and sintered at 900 °C. The black curve shows the experimental data, while the red curve indicates the fit used to evaluate the Ti^{3+} defect concentration.
